# Establishment of a Risk Score Model for Early Prediction of Severe H1N1 Influenza

**DOI:** 10.3389/fcimb.2021.776840

**Published:** 2022-01-04

**Authors:** Siran Lin, YuBing Peng, Yuzhen Xu, Wei Zhang, Jing Wu, Wenhong Zhang, Lingyun Shao, Yan Gao

**Affiliations:** ^1^ Department of Infectious Diseases, Shanghai Key Laboratory of Infectious Diseases and Biosafety Emergency Response, National Medical Center for Infectious Diseases, Huashan Hospital, Fudan University, Shanghai, China; ^2^ Department of Urology, RenJi Hospital Affiliated to Shanghai Jiaotong University School of Medicine, Shanghai, China; ^3^ National Clinical Research Center for Aging and Medicine, Huashan Hospital, Fudan University, Shanghai, China; ^4^ State Key Laboratory of Genetic Engineering, School of Life Science, Fudan University, Shanghai, China; ^5^ Key Laboratory of Medical Molecular Virology (Key Laboratories of the Ministry of Education (MOE)/Key Laboratories of the Ministry of Health (MOH)) and Institutes of Biomedical Sciences, Shanghai Medical College, Fudan University, Shanghai, China

**Keywords:** differentially expressed gene, H1N1, prediction, risk score model, severe influenza

## Abstract

H1N1 is the most common subtype of influenza virus circulating worldwide and can cause severe disease in some populations. Early prediction and intervention for patients who develop severe influenza will greatly reduce their mortality. In this study, we conducted a comprehensive analysis of 180 PBMC samples from three published datasets from the GEO DataSets. Differentially expressed gene (DEG) analysis and weighted correlation network analysis (WGCNA) were performed to provide candidate DEGs for model building. Functional enrichment and CIBERSORT analyses were also performed to evaluate the differences in composition and function of PBMCs between patients with severe and mild disease. Finally, a risk score model was built using lasso regression analysis, with six genes (*CX3CR1*, *KLRD1*, *MMP8*, *PRTN3*, *RETN* and *SCD*) involved. The model performed moderately in the early identification of patients that develop severe H1N1 disease.

## Introduction

H1N1 is one of the most widespread influenza A viruses in humans, which first appeared in Mexico and the United States in April 2009, and brought extensive influenza outbreaks (Garten et al., 2009). The 2009 H1N1 pandemic caused an estimated 250,000 – 500,000 deaths during the first 12 months of global circulation (Dawood et al., 2012). Although patients infected with H1N1 generally show mild symptoms, some patients are severely affected, with viral pneumonia and sometimes multiple organ failure. In clinical practice, patients with severe influenza often miss the best intervention time because physicians cannot tell at an early stage whether the disease will develop in a severe form.

High viral loads and excessive host response are thought to contribute to severe influenza (de Jong et al., 2006; Peiris et al., 2009). Previous studies have revealed that severe disease is often seen among persons aged > 65 years, infants, pregnant women, and individuals of any age with underlying health conditions (Bautista et al., 2010; Hui et al., 2010). In addition, pathways related to interferon, ubiquination, and neutrophils were found to be potential predictors of influenza A disease severity based on transcriptome analysis (Hoang et al., 2014; Dunning et al., 2018). Although transcriptional signatures of mild and severe influenza patients have been clearly analyzed and clarified, there is no model that can be directly applied to predict the severity of the disease. To further use the existing transcriptome data to identify the severity of influenza, we collected two large influenza cohorts and focused on patients infected with H1N1 to build a risk score model.

## Materials and Methods

### Data Collection

The RNA sequence datasets (GSE111368, GSE61821, and GSE101702) were obtained from the GEO DataSets (http://www.ncbi.nlm.nih.gov/geo/). The GSE111368 dataset included blood samples of influenza-infected patients at three time points: at enrolment, approximately 48h after enrolment, and >4 weeks after enrolment. We chose H1N1-infected patients and selected their samples at enrolment for further analysis, which were consisted of 67 patients with severe disease and 27 patients with mild disease that served as the discovery cohort. The dataset GSE61821 included samples of 54 mild and 32 severe H1N1-infected patients at an early stage. Another dataset (GSE101702) had 107 patients with influenza A virus infection, with 63 mild cases and 44 severe cases, and the subtype of the virus was unknown. GSE61821 and GSE101702 were used as the validation cohort.

### Identification of Differentially Expressed Genes (DEGs) and Functional Enrichment Analysis

The expression dataset of the discovery cohort was employed to identify DEGs between severe and mild H1N1 pneumonia patients *via* the R software limma package (Ritchie et al., 2015), with a false discovery rate (FDR) < 0.05 and a log2 |fold change| > 1 as cutoff values. A total of 172 DEGs was selected as shown in the volcano plot by ggplot2. Clustering analyses were performed to show expression patterns of the DEGs, using the pheatmap R package.

Functional enrichment analysis of Gene Ontology (GO), Kyoto Encyclopedia of Genes and Genomes (KEGG), and Gene Set Enrichment Analysis (GSEA) were performed using the ClusterProfiler package (Yu et al., 2012). GO was used to describe gene functions through three aspects: biological process (BP), cellular component (CC), and molecular function (MF). KEGG and GSEA were applied to further illuminate the pathways of the DEGs. P < 0.05 was set as the threshold.

### Estimation of Immune Cell Type Abundances

CIBERSORT is a gene expression‐based deconvolution algorithm (Newman et al., 2015), that uses a leukocyte gene signature matrix consisting of 547 genes, termed LM22, to distinguish immune cell types. Normalized gene expression data of the primary dataset were used to characterize the immune cell composition with CIBERSORT (http://cibersort.stanford.edu/) with the default signature matrix at 1000 permutations. The sum of all estimated immune cell type fractions was equal to 1 for each sample.

### Weighted Correlation Network Analysis (WGCNA)

The gene co-expression network of the primary cohort was obtained with the WGCNA package (Langfelder and Horvath, 2008). A total of 79 out of 94 samples were clustered and used to screen the power function. The power of β = 18 (scale-free R^2^ = 0.80) was chosen to construct a scale-free network using pairwise Pearson correlation analysis. Finally, we counted the disparity of module eigengenes and illustrate the cluster tree of modules, selected the cutting line of the module tree diagram, and merged some modules. The correlation between modules and disease severity was shown by heatmap.

### Model Foundation and Validation

DEGs in modules highly correlated to disease severity were extracted for logistic regression analysis using the lasso method. In this method, a small group of genes associated with disease severity was selected by shrinkage of the regression coefficient *via* imposition of a penalty proportional to their size. We used the glmnet R package to perform lasso regression analysis. First, we randomly selected 70% of the primary dataset as training data and the rest as testing data. Then, the training dataset was used to calculate and select the optimal solution of the parameter λ, which gave the minimum cross-validated error and was then applied to predict the testing data. Finally, a six-gene model was built utilizing the regression coefficients derived from the analysis. The model was used to calculate risk score (risk score = sum of coefficients × normal expression levels of genes) and predict disease severity.

Receiver operating characteristic (ROC) analysis was performed to assess the predictive ability of the prediction model using the pROC R package. First, we used the testing data to compare the accuracies of the risk score model and the age model. Then, to test the applicability of our model, we applied it to the validation cohorts.

## Results

### Different Gene Expression Between Patients With Severe and Mild H1N1 Infection

**Figure 1:**
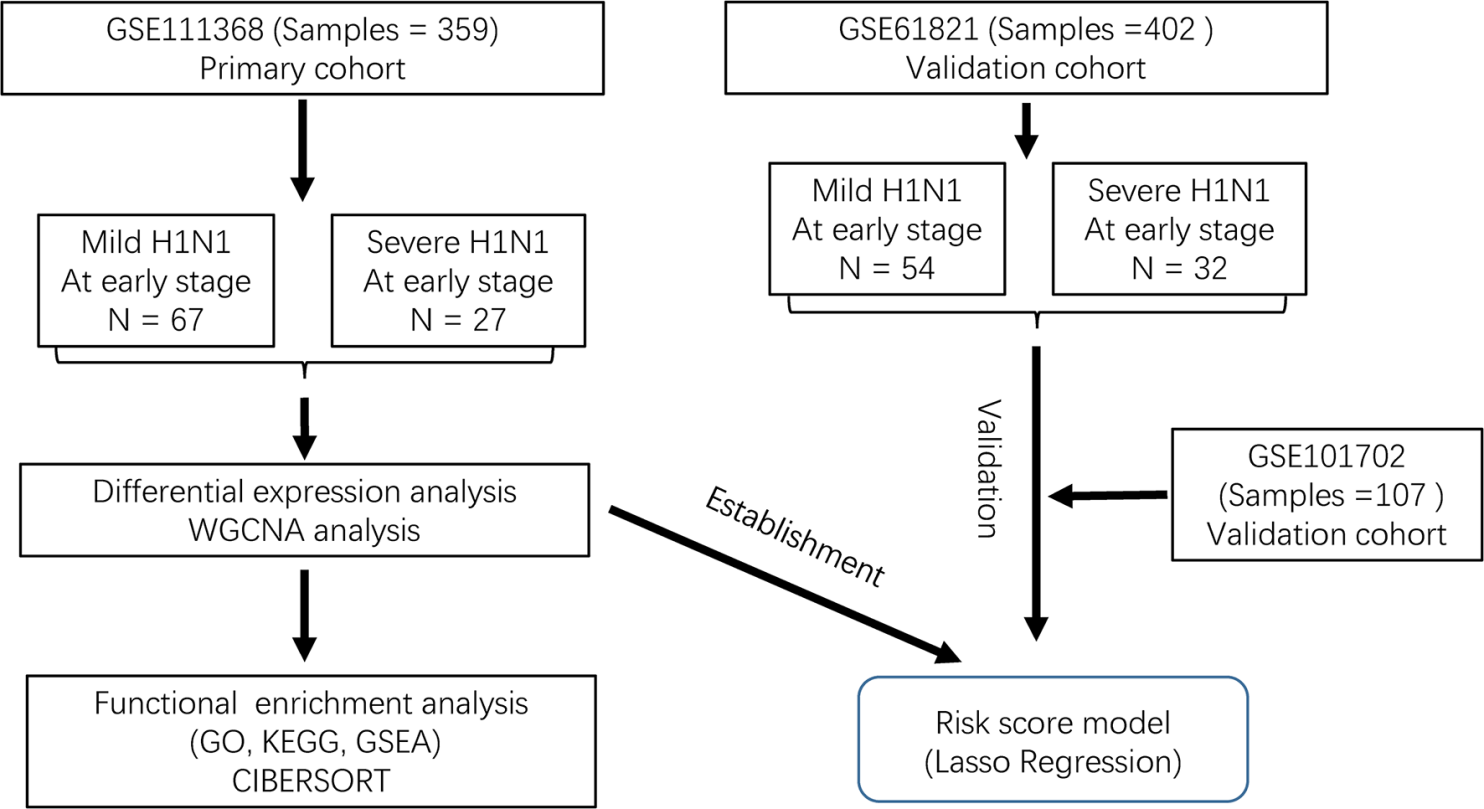
Flow diagram of data collection and analysis.

**Figure 2:**
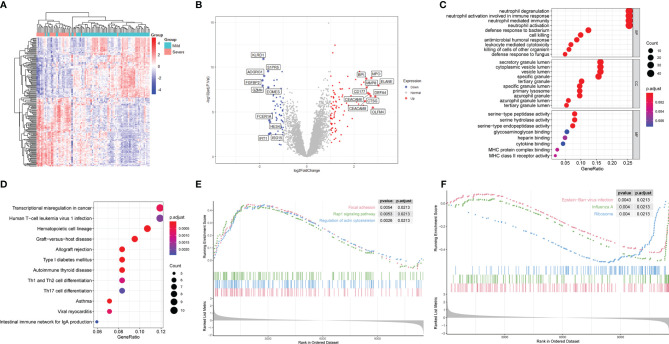
Differential gene expression and functional enrichment analyses of patients with severe and mild H1N1 at an early stage in GSE111368. **(A)** Heatmap of differentially expressed genes (DEGs). **(B)** Volcano map of DEGs. Red and blue spots represent up-regulated and down-regulated genes, respectively. **(C)** DEGs were subjected to GO analysis in BP, CC and MF. **(D)** DEGs were subjected to KEGG analysis. **(E)** Upregulated pathways in GO-GSEA. **(F)** Downregulated pathways in GO-GSEA.

The workflow is shown in **Figure 1**. We used the GSE111368 dataset to identify DEGs, perform functional enrichment analysis, and build the risk score model. Then, the GSE61821 and GSE101702 datasets were used to validate the model. A total of 172 DEGs were identified from the primary dataset, with 105 genes upregulated and 67 genes downregulated in patients with severe disease compared to patients with mild disease. Heatmap analysis by hierarchical clustering showed that the DEGs presented differential expression profiles between patients with severe and mild disease (**Figure 2A**). In addition, the detailed distribution of all the DEGs on the two dimensions of -log10 (FDR) and log2 (FC) were shown through a volcano map (**Figure 2B**), with the top ten upregulated and downregulated genes marked in the figure.

To characterize the biological functions of these DEGs, GO analysis was performed. Enriched GO terms in BP were predominately processes related to immune responses, of which the top four were neutrophil degranulation, neutrophil activation involved in immune response, neutrophil activation and neutrophil mediated immunity (**Figure 2C**). Enriched GO terms in CC revealed that genes involved in formation of different cytoplasmic vesicles and granules were differentially activated in the two groups (**Figure 2C**). Among these granules, secretory vesicle, azurophil granules and specific granules are of great importance in neutrophil function, which contain the proteins necessary to mediate the recruitment, chemotaxis, antimicrobial function, and extracellular traps formation of neutrophils. Only eight GO terms were enriched in MF pathways, of which the top three were serine-type peptidase activity, serine hydrolase activity and serine-type endopeptidase activity (**Figure 2C**).

Different kinds of pathways were enriched in KEGG analysis (**Figure 2D**), of which the immune-related pathways were Th1 and Th2 cell differentiation and Th17 cell differentiation. Moreover, GSEA analysis of KEGG results demonstrated that focal adhesion, regulation of actin cytoskeleton, and Rap1 signaling pathway were the top three upregulated, whereas Epstein-Barr virus infection, influenza A, and ribosome were the top three downregulated pathways (**Figures 2E, F**).

### Estimated Proportions of Immune Cells by CIBERSORT

**Figure 3:**
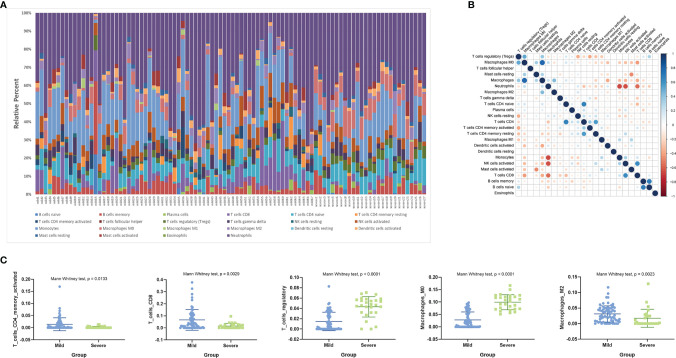
Estimated immune cell fractions using CIBERSORT. **(A)** Relative proportion of immune infiltration in patients with severe and mild H1N1 disease. **(B)** Correlation matrix of all 22 immune cell proportions. **(C)** Box plots visualizing significantly different immune cells between patients with severe and mild H1N1 disease.

Using the CIBERSORT algorithm, we first investigated the difference in immune cell components between severe and mild H1N1-infected patients. **Figure 3A** summarizes the results obtained from 94 H1N1 patients (**Figure 3A**). The proportions of immune cells varied within and between the two groups. In addition, the proportions of different subpopulations of immune cells were weakly to moderately correlated (**Figure 3B**). Patients with severe disease had significantly higher proportions of regulatory T cells and M0 macrophages, and significantly lower proportions of memory-activated CD4+ T cells, CD8+ T cells, and M2 macrophages than patients with mild disease (P < 0.05) (**Figure 3C**).

Functional enrichment analysis by GO and KEGG showed that immune pathways associated with neutrophils were significantly upregulated and activated in patients with severe disease. However, the neutrophil fractions estimated by CIBERSORT showed no significant difference between the two groups. Enrichment of Th1 and Th2 cell differentiation and Th17 cell differentiation pathways by DEGs were associated with different proportions of macrophage and T cell subpopulations between patients with mild and severe disease.

### Gene Modules Analyzed by WGCNA

**Figure 4:**
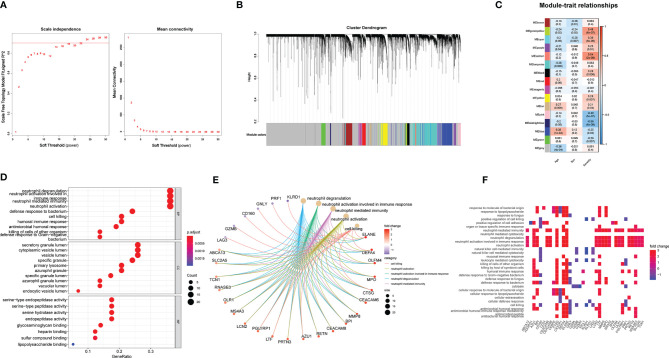
WGCNA is applied to analyze gene modules. **(A)** Scale-free fit index and mean connectivity described for various soft-thresholding powers (β). **(B)** Color-coded co-expression modules constructed in gene dendrogram. **(C)** Module–trait relationships. The meaning of each row refers to the corresponding correlation and p-value. **(D–F)** GO analysis applied in the DEGs extracted from the midnight blue, salmon, pink, and green yellow modules.

To further investigate key genes related to H1N1 disease severity, we performed WGCNA analysis. We selected the soft threshold before constructing WGCNA, as shown in **Figure 4A**. With the soft threshold as 18, gene modules were analyzed among all the genes in the primary dataset. A total of 16 color modules were identified (**Figure 4B**). Most genes were distributed in the gray module, indicating that these genes were not clustered (**Figure 4B**). Then, all color modules were used to analyze the module-trait (disease severity) co-expression similarity and adjacency. The midnightblue, salmon, pink, and greenyellow modules were most related to disease severity (**Figure 4C**). We extracted genes from the four modules and performed GO and GSEA analyses to them. The results of GO analysis were nearly the same as that of all DEGs (**Figure 4D**). In gene networks of enriched GO terms, most genes were associated with neutrophil function and were significantly upregulated (**Figures 4E, F**). Enrichment of endomembrane system, transport, vesicle, extracellular region, and localization shown in the GSEA analysis suggests an active metabolic activity in patients with severe disease (**Supplementary Figure 1**).

### Building and Validation of Risk Score Model

**Figure 5:**
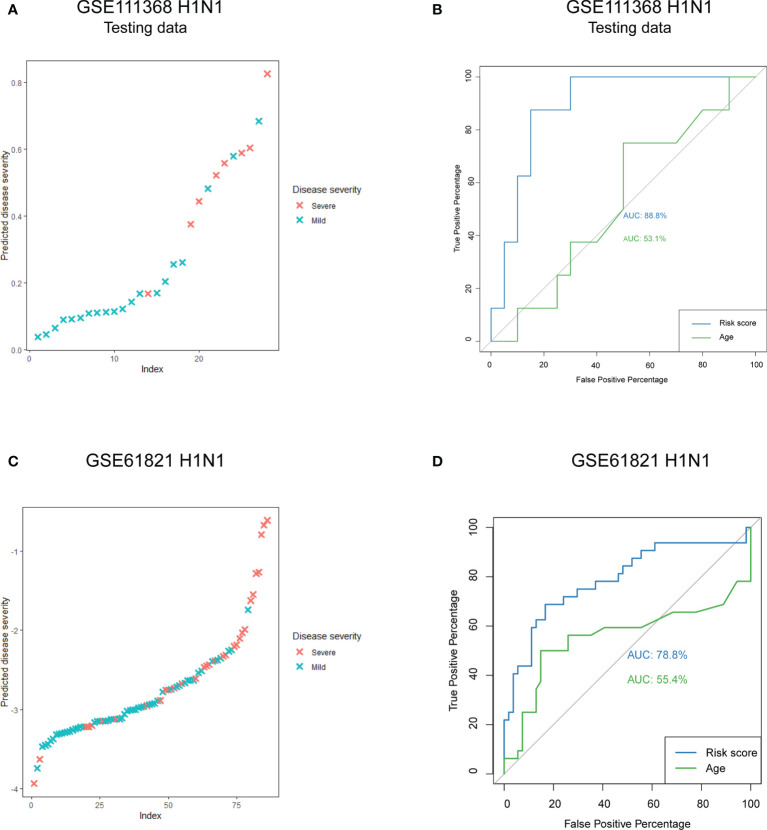
Predictive performance of the risk score model. **(A)** Predicted disease severity of the testing data in the primary cohort. **(B)** ROC curve analyses of the risk score model in the testing data. **(C)** Predicted disease severity of patients in the validation cohort. **(D)** ROC curve analyses of the risk score model in the validation cohort.

A total of 97 DEGs from the WGCNA modules that were most related to disease severity were extracted to construct a risk score model. We chose LASSO regression analysis because it is suitable for constructing models when there are a large number of correlated covariates. Six genes were selected based on the training data: *CX3CR1*, *KLRD1*, *MMP8*, *PRTN3*, *RETN* and *SCD*. The risk score for disease severity = (-1.209) + (-0.132 × normal expression value of *CX3CR1*) + (-0.683 × normal expression value of *KLRD1*) + (0.304 × normal expression value of *MMP8*) + (0.258 × normal expression value of *PRTN3*) + (0.145 × normal expression value of *RETN*) + (0.023 × normal expression value of *SCD*). We calculated the risk score for each patient in the testing data and compared it to their real disease severity (**Figure 5A**). The risk score could clearly distinguish patients with mild and severe disease (**Figure 5A**). Furthermore, we measured the predictive performance of the model using ROC curves. The area under the ROC (AUC) value was 88.8% for the risk score model, which was rather better than age at 53.1% (**Figure 5B**). To determine whether the risk score model was robust, we assessed the performance of the model in the validation cohort GSE61821 (**Figure 5C** and 5D). The AUC value for the validation cohort was 78.8% compared to 55.4% for age (**Figure 5D**).

**Figure 6:**
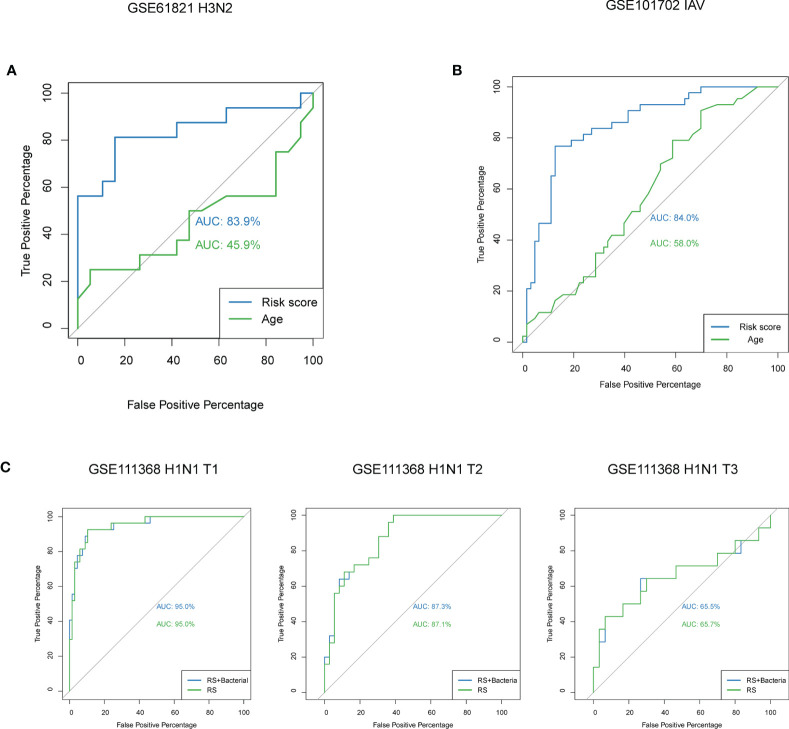
Predictive performance of the model in patients with other types of influenza and the effect of bacterial coinfection and time on the model. **(A)** ROC curve analyses of the risk score model for patients with H3N2 from GSE61821. **(B)** ROC curve analyses of the risk score model for patients with influenza A virus infection from GSE101702. **(C)** ROC curve analyses of the risk score model with and without the parameter of bacterial coinfection at different time points. IAV, influenza A virus; T1, at enrolment; T2, approximately 48h after enrolment; T3, and >4 weeks after enrolment.

In order to identify whether the model could be used in other types of influenza, we calculated the risk scores for H3N2 patients from GSE61821 dataset, and the AUC value was 83.9% for our model (**Figure 6A**). In addition, we also performed prediction for patients with influenza A virus infection from another dataset GSE101702, and the AUC value was 84.0% for our model (**Figure 6B**). To determine whether bacterial coinfection had an impact on the accuracy of the model, we added the clinical parameter of bacterial coinfection to our model to reanalyze. As shown in **Figure 6C**, the AUC values for our model were not affected by the parameter (95.0% vs. 95.0%). We also compared the predictive effectiveness of the model in different time points, and we found it had a better performance in the early stage of disease (**Figure 6C**).

## Discussion

Statistical models like machine learning trained with high-throughput expression data have long been employed to identify molecular signatures in cancer. However, not many studies have used this method in infectious diseases, and we are the first to employ it on influenza. To find the key genes related to the severity of H1N1 disease, we did both DEG and WGCNA analyses. Then, DEGs were extracted from the WGCNA modules associated with disease severity and were used for lasso regression to obtain a risk score model of six genes. The proposed model was also validated, and ROC curve results suggest that the model was accurate in identifying patients who may develop severe influenza. We also performed functional enrichment and CIBERSORT analyses to provide functionally relevant evidence for the selected genes. Functional enrichment analysis showed that multiple immune-related pathways were expressed differently in the mild and severe groups, especially those related to neutrophils. This is also reflected in the selected genes, of which four are related to immune responses.

Among the six genes, the one with the largest weight for risk score was *KLRD1*. *KLRD1* encodes NK cell receptor CD94 and forms a heterodimer with NKG2 on NK cells and a small portion of CD8+ T cells (Gunturi et al., 2004). The CD94/NKG2 receptor functions as an inhibitor or an activator depending on which isoform of NKG2 is expressed (Glienke et al., 1998; Martinet and Smyth, 2015). The CD94/NKG2 receptor regulates the effector function and cell survival of NK and CD8+ T cells, thereby playing a key role in the innate and adaptive immune responses to pathogens. As shown in previous studies, most CD8+ T cells and NK cells expressing high levels of CD94 co-express the inhibitory subtype NKG2A, which plays a role in blocking cytotoxicity and simultaneously protects the cells from apoptosis (Gunturi et al., 2004). Daniel R. Ram et al. have analyzed the baseline immunization spectrum of volunteers before vaccination against influenza and found that, after vaccination, the expression levels of *KLRD1* in the peripheral blood of symptomatic volunteers was lower than that of asymptomatic patients (Bongen et al., 2018). The baseline expression level of *KLRD1* was negatively correlated with the severity of symptoms. In the results of our analysis, the expression levels of *KLRD1* in patients with mild disease was significantly higher than that in patients with severe disease, which is consistent with Daniel R. Ram’s study. In addition, the results of CIBERSORT revealed a higher level of CD8+ T cells in patients with mild disease. The high expression levels of *KLRD1* may help effector CD8+ T cells to survive and thus protect against influenza. However, because our study did not observe changes in the expression levels of NKG2, we cannot determine whether the high expression levels of *KLRD1* activate or inhibit the function of NK cells.

The expression of both *KLRD1* and *CX3CR1* expression was inversely related to H1N1 disease severity. CX3CR1 is expressed on the surface of a variety of immune cells, including myeloid cells such as monocytes, macrophages, and microglia, as well as terminally differentiated effector CD8+ T cells and NK cells (Harrison et al., 1998; Nishimura et al., 2002). In the blood, CX3CR1 expression is restricted to monocytes (Geissmann et al., 2003) and mediates the migration and adhesion of monocytes through interaction with its ligand CX3CL1, which is necessary for the rapid accumulation of monocytes to dangerous sites to trigger early immune responses (Imai et al., 1997; Imai and Yasuda, 2016; Hamon et al., 2017). In addition, the CX3CL1-CX3CR1 complex activates the NF-κB or cyclic adenosine monophosphate response element binding protein signaling pathways, promoting the secretion of inflammatory cytokines (Sheridan and Murphy, 2013). Studies have shown that CX3CR1 plays an important role in the function of memory CD8+ T cells (Böttcher et al., 2015; Gerlach et al., 2016). CX3CR1+CD8+ T cells co-express cytotoxic effect molecules (granzyme B and perforin) and show strong cytotoxicity (Böttcher et al., 2015). Virus-specific memory CX3CR1+CD8+ T cells increases when infections are controlled spontaneously or through therapeutic intervention, but are present in small numbers in chronic infection states (Böttcher et al., 2015; Desai et al., 2018). In our results, the CX3CR1 expression levels in patients with severe H1N1 is significantly lower than those in patients with mild disease. This result suggests that the dysfunction of blood monocytes and CD8+ T cells at an early stage may be related to the worsening of the disease after H1N1 infection.

The other four genes are positively correlated with H1N1 disease severity, and the proteins encoded by *MMP8* (matrix metalloproteinase-8) and *PRTN3* (proteinase 3) are related to neutrophil function. MMP8, also called collagenase 2 or neutrophil collagenase, is stored as an inactive enzyme in neutrophil granules, which can be quickly released to the site of inflammation after neutrophil activation. With its unique characteristics, MMP8 has been shown to play an important role in the pathogenesis of respiratory diseases such as acute respiratory distress syndrome or acute lung injury (Fligiel et al., 2006), COPD (Vernooy et al., 2004), interstitial lung disease (Choi et al., 2002), and hospital-acquired pneumonia (Schaaf et al., 2008). First, MMP8 is the most potent collagenase to degrade collagen type I, a major extracellular matrix component of the lung (Hasty et al., 1987). Second, MMP8 is highly sensitive to reactive oxygen species, which are often associated with lung disorders (Saari et al., 1992). In patients, the upregulation of MMP8 expression is often related to the progression of inflammatory diseases. These results are also consistent with our conclusions that high expression levels of MMP8 increased the risk of H1N1 patients to develop severe disease. PRTN3 is a neutrophil serine protease family member, which is produced during neutrophil development in the bone marrow and stored in azurophil granules of mature neutrophils. It plays a critical role in killing bacteria and post-translational modification of cytokines, thereby mediating damage to tissues (Kalupov et al., 2009; Almansa et al., 2012). Higher expression levels of *MMP8* and *PRTN3* in patients with severe disease are consistent with the activation of neutrophil-related immune pathways in the GO and GSEA analyses.

Both resistin (RETN) and stearoyl-CoA desaturase (SCD) are molecules that regulate lipid metabolism, and there are few studies on them in infectious diseases. Limited studies have shown that RETN is positively related to disease severity of sepsis (Sundén-Cullberg et al., 2007; Koch et al., 2009), in which circulating leukocytes increase the expression levels of resistin, and the latter could contribute to disease severity by regulating the expression levels of ICAM-1 and VCAM-1 in vascular endothelial cells (Macdonald et al., 2014; Macdonald et al., 2017). In addition, Lauren Miller et al. showed that resistin inhibits the oxidative burst of neutrophils by partially reducing the polymerization of F-actin to block its bactericidal activity without affecting macrophages or monocytes (Miller et al., 2019). Therefore, the role of RETN in H1N1 infections may be the combination of its regulation on different kinds of immune cells. SCD is a key rate-limiting enzyme in the synthesis of unsaturated fatty acids with two isoforms (1 and 5) having been identified (Kim and Ntambi, 1999; Wang et al., 2005). By regulating the balance between saturated and unsaturated fatty acids, SCD plays an essential role in a variety of cellular processes, including lipid synthesis, hormonal signaling, and inflammation (Strable and Ntambi, 2010; Sampath and Ntambi, 2014; ALJohani et al., 2017). Recent studies have demonstrated that unsaturated fatty acids play critical roles in hepatitis C and dengue virus replication (Nguyen et al., 2014; Gullberg et al., 2018) and an SCD1 inhibitor strongly suppresses the replication of these viruses (Nio et al., 2016; Hishiki et al., 2019). Our analysis shows that the high expression levels of SCD are related to the aggravation of H1N1 disease, but the correlation coefficient was not high. Further research is still needed to determine whether SCD is also involved in the life cycle of the H1N1 virus.

In summary, our analysis of two influenza datasets demonstrate that neutrophil-mediated immune response may play an important role in the progression of severe pneumonia caused by H1N1 infection. The risk score model based on the expression levels of *CX3CR1*, *KLRD1*, *MMP8*, *PRTN3*, *RETN*, and *SCD* is a reliable prediction model for early identification of patients with severe H1N1 disease. And it has the potential to be used in patients with H3N2 infection.

## Data Availability Statement

The original contributions presented in the study are included in the article/Supplementary Material. Microarray data that support the findings of this study are available at Gene Expression Omnibus (Accession numbers: GSE111368, GSE61821, and GSE101702); [https://www.ncbi.nlm.nih.gov/geo/]. Further inquiries can be directed to the corresponding author.

## Author Contributions

SL, YP, and YG contributed to the conception and design of the study. SL, YX, WZ (Wei Zhang), and JW performed data collection, analysis and/or visualization. SL wrote the first draft of the manuscript and YP wrote sections of the manuscript. WZ (Wenhong Zhang), LS, and YG revised the manuscript. All authors contributed to the article and approved the submitted version.

## Funding

This work was supported by the Key Laboratory Project of Shanghai Science and Technology Commission [grant number 20dz2210400].

## Conflict of Interest

The authors declare that the research was conducted in the absence of any commercial or financial relationships that could be construed as a potential conflict of interest.

## Publisher’s Note

All claims expressed in this article are solely those of the authors and do not necessarily represent those of their affiliated organizations, or those of the publisher, the editors and the reviewers. Any product that may be evaluated in this article, or claim that may be made by its manufacturer, is not guaranteed or endorsed by the publisher.
