# Supplementary figures and images for: Establishment of a Risk Score Model for Early Prediction of Severe H1N1 Influenza
Source: Front Cell Infect Microbiol. 2022 Jan 4;11:776840. doi: 10.3389/fcimb.2021.776840 (PMC8764189; doi:10.3389/fcimb.2021.776840)

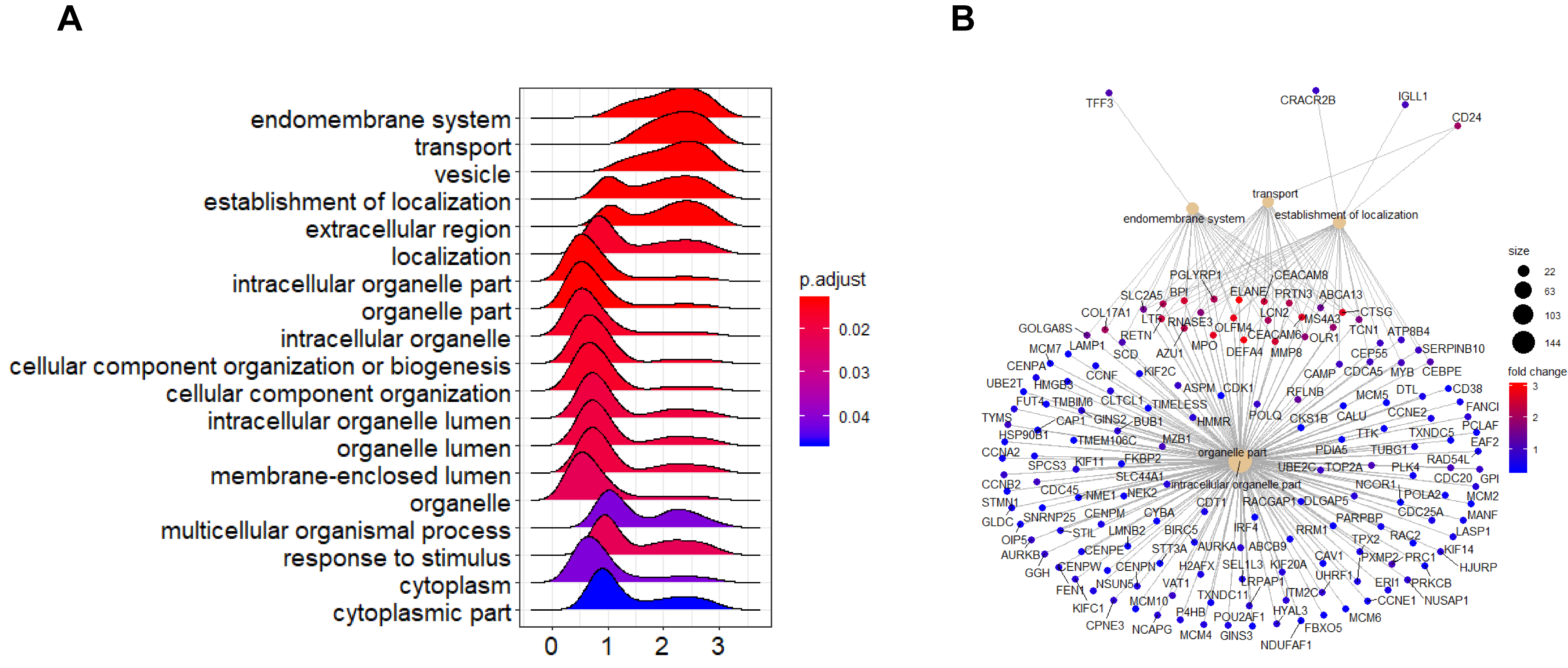

Supplement: Supplementary file 1 [file Image_1.tif]
